# Infectious mononucleosis

**DOI:** 10.1038/cti.2015.1

**Published:** 2015-02-27

**Authors:** Henry H Balfour, Samantha K Dunmire, Kristin A Hogquist

**Affiliations:** 1 Department of Laboratory Medicine and Pathology, University of Minnesota Medical School, Minneapolis, MN, USA; 2 Department of Pediatrics, University of Minnesota Medical School, Minneapolis, MN, USA

## Abstract

Infectious mononucleosis is a clinical entity characterized by pharyngitis, cervical lymph node enlargement, fatigue and fever, which results most often from a primary Epstein–Barr virus (EBV) infection. EBV, a lymphocrytovirus and a member of the γ-herpesvirus family, infects at least 90% of the population worldwide, the majority of whom have no recognizable illness. The virus is spread by intimate oral contact among adolescents, but how preadolescents acquire the virus is not known. During the incubation period of approximately 6 weeks, viral replication first occurs in the oropharynx followed by viremia as early as 2 weeks before onset of illness. The acute illness is marked by high viral loads in both the oral cavity and blood accompanied by the production of immunoglobulin M antibodies against EBV viral capsid antigen and an extraordinary expansion of CD8^+^ T lymphocytes directed against EBV-infected B cells. During convalescence, CD8^+^ T cells return to normal levels and antibodies develop against EBV nuclear antigen-1. A typical clinical picture in an adolescent or young adult with a positive heterophile test is usually sufficient to make the diagnosis of infectious mononucleosis, but heterophile antibodies are not specific and do not develop in some patients especially young children. EBV-specific antibody profiles are the best choice for staging EBV infection. In addition to causing acute illness, long-term consequences are linked to infectious mononucleosis, especially Hodgkin lymphoma and multiple sclerosis. There is no licensed vaccine for prevention and no specific approved treatment. Future research goals are development of an EBV vaccine, understanding the risk factors for severity of the acute illness and likelihood of developing cancer or autoimmune diseases, and discovering anti-EBV drugs to treat infectious mononucleosis and other EBV-spurred diseases.

Infectious mononucleosis is the name coined by Sprunt and Evans in 1920^1^ for an acute infectious disease consisting of fever, cervical lymphadenopathy and pharyngitis accompanied by atypical large peripheral blood lymphocytes. Its major cause is Epstein–Barr virus (EBV). We now know that the characteristic atypical lymphocytes, carefully described morphologically by Downey and McKinlay,^2^ are actually activated CD8^+^ T cells,^3^ which are responding to EBV-infected B cells.^4^ Infectious mononucleosis represents a significant health risk because of the severity and duration of the acute illness and also because of the potential for long-term complications in the form of certain cancers and autoimmune diseases.

## Identification of EBV as the cause of infectious mononucleosis

Infectious mononucleosis was recognized as a unique disease in the 1880s by Nil Filatov, a Russian pediatrician, who called the syndrome ‘idiopathic adenitis.^5^ Indeed, its etiology remained a mystery until 1967 when a serendipitous event established the causal relationship between infectious mononucleosis and EBV.

EBV was discovered by Epstein *et al.*^6^ in 1964 using electron microscopy to detect the virus in cultured Burkitt lymphoma cells. Epstein believed that another laboratory should repeat his finding, but British virologists were not interested in collaborating.^7^ ‘As a last resort,' Epstein sent the Burkitt cells to Klaus Hummeler in Philadelphia, who had just spent a sabbatical with Epstein.^8^ As Hummeler's laboratory had been recently dismantled because of lack of funds, he brought the cells to the Henle laboratory, which was also in Philadelphia, where Epstein's discovery of a new herpesvirus was quickly confirmed,^9^ and additional studies launched to further characterize this virus.

Now comes a truly ‘once-upon-a-time' story. A technologist working in the Henle laboratory who lacked antibodies against EBV regularly donated lymphocytes for EBV transmission/transformation experiments but her cells never survived in culture.^8, 10^ She became ill in August 1967 and missed 5 days of work. Her physician's clinical impression was rubella versus infectious mononucleosis. Her rubella antibodies were negative but her heterophile antibody test, which had been established as the laboratory method of choice to diagnose infectious mononucleosis,^11^ was positive. Her lymphocytes now grew continuously in culture and were positive for EBV antigens. She also had acquired EBV-specific antibodies, which was the crucial clue that EBV was responsible for a common acute infectious disease. Additional serum samples were obtained. Especially valuable were those from researchers at Yale, who had amassed a prospective serum bank from sick students and thus had pre- and post-illness samples. These were ideal reagents to prove conclusively that primary EBV infection caused infectious mononucleosis.^12^

## Epidemiology of infectious mononucleosis

EBV infection among adolescents and young adults is spread primarily by deep kissing as documented by Hoagland's clinical observations,^13^ and confirmed many years later by a prospective study among undergraduate university students.^14^ Sexual intercourse has been purported to enhance transmission,^15^ but our University of Minnesota study found that subjects who reported kissing with or without penetrative sexual intercourse had the same higher risk of primary EBV infection throughout the undergraduate years as compared with subjects who reported no kissing and no sex.^14^

In unusual circumstances, primary EBV infection can also be transmitted by blood transfusion,^16^ solid organ transplantation^17^ or hematopoietic cell transplantation.^18^ For instance, Alfieri *et al.*^19^ used polymorphisms in the EBV *BAM*HI-K fragment length and EBV nuclear antigen (EBNA) -1, -2 and -3 proteins to identify the specific blood donor who transmitted EBV to a 16-year-old liver transplant recipient. That recipient subsequently developed infectious mononucleosis.

How preadolescent children contract EBV is unknown. It could be that they are infected by their parents or siblings who shed EBV periodically into their oral secretions.^20^ A graphic illustration of this is the acquisition of EBV by Melanesian infants whose multiple caregivers chew the food themselves before giving it to the baby.^21^

The incubation period of infectious mononucleosis has been observed to be between 32 and 49 days.^13^ A well-documented Swedish case was reported in which the kissing event occurred 38 days before onset of symptoms.^22^ Behavioral, virologic and immunologic data collected during prospective studies in university undergraduates point to a modal incubation period of 42 days (Balfour *et al.*, unpublished observations).

## Clinical manifestations of the acute illness

Infectious mononucleosis is a clinical entity characterized by pharyngitis, cervical lymph node enlargement, fatigue and fever. The disease occurs worldwide with no seasonal predilection. It is recognized most frequently in adolescents and young adults from developed countries for reasons that are not completely understood. Part of the explanation is lack of recognition of the syndrome in preadolescents. The heterophile antibody test is often unreliable in young children, particularly those under 4 years of age. Thus, assays specific for EBV must be performed in these cases, lest the diagnosis of infectious mononucleosis be missed.^23^ Infectious mononucleosis in preadolescents is not rare. As a pediatrician, one of us (HHB) has seen numerous cases in children younger than 12 years old. Indeed, infectious mononucleosis was first described by a Russian pediatrician.^5^

A second reason could be that deep kissing transmits a large amount of infectious virus. In contrast, young children probably acquire the virus from asymptomatic parents or siblings who shed low levels of EBV in their oral secretions and transmit a smaller infectious inoculum. Parents of young children (<6 years of age) have EBV in their oral secretions about 30% of the time but the median quantity is only 4900 copies ml^−1^ (Cederberg *et al.*, unpublished observations). In contrast, during the acute and convalescent stages of primary EBV infection, young adults shed a median of 63 100 copies ml^−1^.^14^

A third possibility is that infectious mononucleosis in adolescents may reflect the response of cross-reactive memory CD8^+^ T cells. For example, influenza-specific CD8^+^ T cells may cross-react with EBV.^24^ As adolescents are presumably more likely to have high numbers of influenza-specific CD8^+^ T cells as compared with young children who have seen relatively few different influenza types, the adolescents would react more strongly against EBV. However, we did not find any evidence of influenza–EBV dual specific CD8^+^ T cells in our cohort^25^ and thus it remains questionable whether preexisting (cross-reactive) CD8^+^ T-cell immunity to EBV would influence the severity of primary EBV infection.

Recent data implicate certain classes of natural killer (NK) cells as important factors in the early control of EBV. Azzi *et al.*^26^ and colleagues detected significantly higher levels of CD56^dim^ NKG2A^+^ killer-cell immunoglobulin-like receptors (KIR)^−^ NK cells in the peripheral blood of children as compared with either adolescents or adults. These findings suggest that differences in this preexisting NK cell population may affect the course of subsequent infection, and may provide an explanation for why infectious mononucleosis occurs more frequently in adolescents and adults than in children.

A final point is that infectious mononucleosis is more commonly seen in developed countries because the age at acquisition of primary EBV infection is older than it is in the developing world.^27^

**Figure 1:**
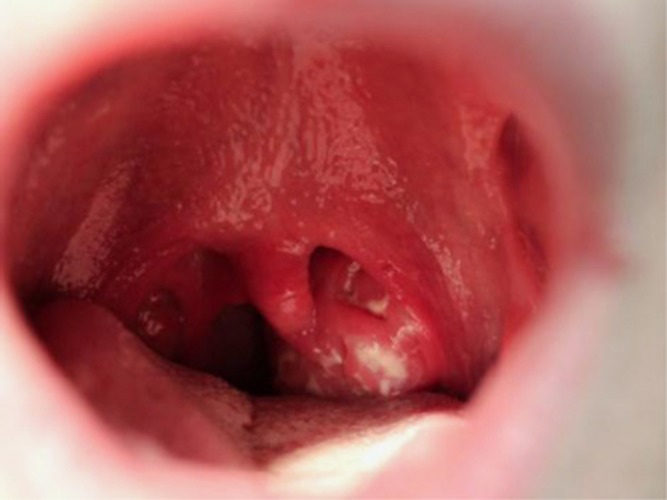
Pharyngitis demonstrating exudative tonsillitis and an enlarged uvula in a 19-year-old undergraduate university student 5 days after onset of infectious mononucleosis. In addition to pharyngitis, he felt febrile, had cervical lymphadenopathy, fatigue and loss of appetite. His sore throat lasted for 9 days and he was fatigued for 29 days.

Most young adults develop infectious mononucleosis after primary EBV infection.^14^ There are two typical clinical presentations. One is the sudden onset of sore throat (Figure 1). Patients also complain of a swollen neck that reflects cervical lymph node enlargement. Another typical presentation is the slow development of malaise, myalgia and fatigue. The most frequent signs and symptoms are: sore throat (95%), cervical lymphadenopathy (80%), fatigue (70%), upper respiratory symptoms (65%), headache (50%), decreased appetite (50%), fever (47%) and myalgia (45%).^14^ Most findings last 10 days or less but fatigue and cervical lymphadenopathy often persist for at least 3 weeks. Other clinical findings, seen in the minority of cases, include abdominal pain, hepatomegaly, splenomegaly, nausea, vomiting, palatal petechiae, periorbital and eyelid edema. Hepatitis occurs in 75% of patients but is usually subclinical (elevation of alanine aminotransferase levels without jaundice or abdominal pain). Rash is not usually seen except in patients given penicillin derivatives, in which case it results from transient penicillin hypersensitivity.^28^ As an apt example, the technologist in the Henle laboratory who provided the major clue that EBV caused infectious mononucleosis had been given ampicillin. Her rash prompted clinicians to think of rubella as well her correct diagnosis, infectious mononucleosis.^10^

## Complications of the acute illness

Serious complications during the acute phase of primary EBV infection are rare. Complications that occur in at least 1% of patients are: airway obstruction because of oropharyngeal inflammation, streptococcal pharyngitis, meningoencephalitis, hemolytic anemia and thrombocytopenia.^29, 30, 31^ Splenic rupture occurs in <1% in patients but is the most feared complication, which sometimes keeps athletes out of competition for weeks.^32^ A reasonable recommendation is that athletes may resume contact sports after 3 weeks of illness as long as they have no ongoing signs or symptoms of acute EBV infection.^33^

## Dynamics of the infection and immune response

During the 6-week incubation period of primary EBV infection, viral replication is first detected in the oral cavity.^14^ There EBV infects both B cells and tonsillar epithelial cells.^34^ Interestingly, the infection efficiency of EBV for these cell types varies depending on the cell type supporting viral replication. *In vitro* studies have shown that virus derived from epithelial cells is better able to infect B cells and vice versa.^35^ Therefore, EBV infection in the oral cavity is likely affected by the cyclic pattern of this switch tropism.

The virus transitions from the oral cavity to the peripheral blood at some point during the incubation period. How and when this transition takes place is not well understood, although copies of the EBV genome can be detected in peripheral blood up to 2 weeks before onset of symptoms (Dunmire *et al.*, unpublished observations). In addition, gene expression profiling has revealed that 2 weeks before symptom onset a systemic type I interferon response can be detected in some individuals who subsequently present with infectious mononucleosis.^36^

The onset of the acute illness is marked by high viral loads in both the oral cavity and blood. This is accompanied by the production of immunoglobulin M (IgM) antibodies against EBV viral capsid antigen (VCA) and an extraordinary expansion of CD8^+^ T lymphocytes.^14^ The response of these CD8^+^ T cells is of particular interest because these cells are important for controlling EBV, a role supported by the fulminant disease that occurs in patients with defects in the function of their T cells, such as the ability to interact and kill EBV-infected B cells.^37, 38^

Acute infectious mononucleosis is characterized by abnormally high numbers of circulating CD8^+^ T cells. Of these cells, many are specific for EBV antigens derived from the immediate early and early stages of lytic infection with a marked bias toward the immediate early stage. Late lytic antigens also generate a specific CD8^+^ T-cell response as revealed by comparing T-cell clones from infectious mononucleosis patients with those of long-term carriers.^39^ In addition to proteins encoded by EBV in the lytic phase, CD8^+^ T cells respond to latent antigens, especially EBNA-2 and EBNA-3.^40, 41^ Thus, the T-cell response is directed at both lytic and latent infections in carriers.

Although it has been established in the literature that numbers of CD4^+^ T cells are not substantially increased during infectious mononucleosis, data exist to support the concept that CD4^+^ T cells are important contributors to the control of EBV. Indeed, CD4^+^ T cells have been shown to recognize several lytic antigens through use of major histocompatibility complex (MHC) II tetramers. These cells are not only present during acute infection, but are maintained in the peripheral blood, albeit at low levels.^42^

**Figure 2:**
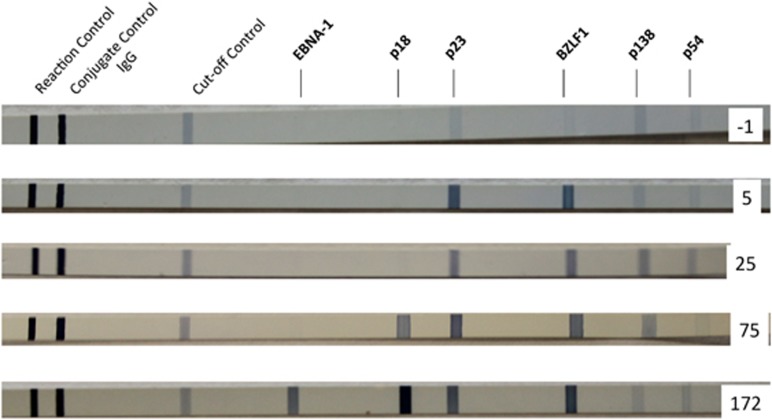
Line immunoblots demonstrating IgG antibody responses to six EBV proteins at five timepoints from the day before onset of illness (day −1) to 172 days postonset. Band intensities equal to or greater than the cutoff control are considered to indicate a specific antibody response. The first antibodies to appear are directed against BZLF1 (immediate early), and p23 (VCA). Antibodies to p138 and p54 (EA) developed next, followed by p18 (VCA) antibodies. Antibodies against EBNA-1 were seen only on the serum sample collected 172 days after onset of illness.

The kinetics of the IgG antibody responses to various EBV proteins are quite distinct as illustrated by the line blot assay (Mikrogen, Neuried, Germany) in Figure 2. This assay contains 6 EBV antigens, 2 of which are components of the VCA structural protein: p23 (BLRF2) and p18 (BFRF3).^43^ IgG antibodies against EBV VCA are usually detectable after the first week of illness and persist for life. The immune response to the p23 component of VCA develops sooner than the response to p18. Three of the EBV antigens belong to the temporal classes of lytic gene products: immediate early, early, and late. Antibodies directed against the immediate EA BZLF1 also appear quickly and remain. Antibody responses to the EAs p138 and p54 are more variable. Although they can be found early after infection, they often become undetectable after convalescence. In contrast, antibodies against EBNA-1, which is a latent gene product, are slow to develop and are usually not detected until 3 months or longer after onset of illness.^44^ However, once they are found they remain present for life. The delayed EBNA-1 antibody response has been shown to correlate with a delayed CD4^+^ T-cell response to EBNA-1.^42^

Although CD8^+^ T cells are recognized as vital factors in the control of EBV infection, NK cells are increasingly being acknowledged as important during infectious mononucleosis, as evidenced by the severe EBV-related outcomes that occur in several immunodeficiencies involving T and NK cells and/or their cytolysis pathways.^45, 46^ Other data also support a role for NK cells during infectious mononucleosis, such as the observation that NK cells *in vitro* preferentially kill EBV-infected cells when the virus transitions into the lytic phase.^47^

Humanized mouse models have more recently allowed for examination of interactions between NK cells and EBV *in vivo*. One such model, the non-obese diabetic *scid* gamma mouse is created by reconstituting immune compartments with CD34^+^ lin^−^ hematopoietic stem cells.^48, 49^ These mice are then infected with the B95.8 strain of EBV and monitored for signs of infectious mononucleosis-like disease such as CD8 lymphocytosis and EBV viremia. Animals depleted of NK cells were found to have more severe signs of EBV-related disease.^50^ In regard to this study, it is of particular interest that depletion of NK cells following initial establishment of EBV infection in non-obese diabetic *scid* gamma mice did not have a significant effect. Given differences in the response to EBV that may be observed between NK cells derived from the tonsil and NK cells derived from the peripheral blood, it seems likely that NK cells have a more prominent role in controlling early infection in the oropharynx than they do in the peripheral blood during the viremic phase.^51^

The importance of peripheral blood NK cells in humans during infectious mononucleosis remains a point of contention as studies have yielded conflicting results. In one such study, an inverse correlation was found between blood virus and the number of NK cells detected in the periphery,^52^ while another larger study found a positive correlation.^14^ Thus, the relevance of NK cells to levels of peripheral blood virus at the viremic stage of infection requires additional investigation.

It is probable, however, that total NK cell numbers may not accurately represent the contribution of this cell type to combating infection. Deeper probing into individual NK subsets in other infections has shown that certain types of NK cells have a more potent effect than others. For example, during primary cytomegalovirus infection, NKG2C^+^ NK cells become expanded and have been demonstrated to respond specifically to that virus.^53^ Although NKG2C^+^ NK cells numbers are not affected by primary EBV infection, NKG2A^+^ NK cells can be detected at greater frequency in the peripheral blood of infectious mononucleosis patients.^54^ Larger numbers of NKG2A^+^ CD54^+^ NK cells are also found in the tonsils of EBV carriers than in non-carriers.^55^ Furthermore, CD56^dim^ NKG2A^+^ KIR^−^ NK cells have been shown to preferentially proliferate in response to EBV-infected cells, as reported in a study of pediatric infectious mononucleosis.^26^

The importance of NK cells is also highlighted by the fact that the virus has a mechanism to hinder NK cell activation during viral replication. The protein product of the EBV open reading frame BZLF1 encodes a peptide sequence that can bind the non-classical MHC-I molecule human leukocyte antigen (HLA)-E. In turn, HLA-E can then engage the inhibitory receptor NKG2A.^56^ Interestingly, an interference mechanism targeted toward adaptive immune cells may have evolved to avoid NK cell surveillance. BILF1 downregulates expression of MHC class I molecules, namely HLA-A and HLA-B, but interestingly does not affect HLA-C, which is inhibitory to NK cells.^57^

In addition, there exists evidence that the transformation of B cells by EBV is limited by the presence of certain types of NK cells. Experiments performed *in vitro* showed that CD56^bright^ CD16^−^ NK cells were preferentially primed by dendritic cells matured by exposure to EBV. Incubation of these NK cells with B cells in the presence of virus resulted in lower B-cell transformation in an interferon-γ-dependent manner. It is worth mentioning that NK cells derived from tonsillar tissue were much more efficient than those isolated from peripheral blood,^51, 58^ which is especially relevant given that numbers of CD56^bright^ CD16^−^ NK cells are drastically reduced in the peripheral blood during infectious mononucleosis.^54^ Thus, NK cells may serve to help control EBV infection in two ways: through direct cytolysis of infected cells and through blockade of transformation via interferon-γ.

During convalescence (3 to 6 months after onset of infectious mononucleosis), CD8^+^ T-cell and NK cell numbers return to normal levels.^14^ It was previously proposed that herpesvirus infection during childhood conveys an advantage to the host by ‘priming' the immune system to better combat subsequent threats. These suppositions were supported by data showing that mice infected with the murine gamma herpesvirus-68 could subsequently more rapidly clear a bacterial challenge,^59^ although these effects were later demonstrated to be transient.^60^ A study in human subjects provided corroborating evidence in the form of gene expression data from peripheral blood mononuclear cells. No long-term gene expression changes were found after primary EBV acquisition, suggesting that the immunological steady state, at least in the periphery, is not appreciably altered following herpesvirus infection.^36^

## Diagnosis of infectious mononucleosis because of primary EBV infection

The cause of infectious mononucleosis cannot be determined on clinical grounds alone. There is a practical way and a precise way to diagnose infectious mononucleosis because of primary EBV infection. The practical way is to obtain laboratory confirmation using a heterophile antibody test. This assay has been used as the standard point-of-care laboratory method ever since its discovery by Paul and Bunnell in 1932.^11^ Paul and Bunnell defined heterophile antibodies as ‘having the capacity to react to certain antigens, which are quite different from, and phylogenetically unrelated to the one instrumental in producing the antibody response.' Heterophile tests use mammalian erythrocytes from various species to detect IgM class antibodies against them, which are raised during the generalized immune upregulation that accompanies acute primary EBV infection.

Heterophile tests are a practical method for confirming the clinical diagnosis. However, they do have drawbacks. Approximately 40% of children 4 years of age or younger do not develop heterophile antibodies following a primary EBV infection.^23^ If the heterophile is the only test ordered, the diagnosis will be missed. Second, heterophile antibodies by definition are not specific and may be present in infections caused by other pathogens, malignancies and autoimmune diseases.^61, 62^ Finally, heterophile antibodies can persist for a year or more and therefore are not always diagnostic of an acute EBV infection.^63^

The most useful specific antibody tests are VCA IgM, VCA IgG and EBNA-1 IgG usually measured using an enzyme immunoassay platform. VCA IgM antibodies are present in 75% of patients during the acute illness.^14^ However, false-positive results have been reported especially with cytomegalovirus infection.^64^ All patients with infectious mononucleosis develop IgG antibodies to VCA,^14^ so this is the best laboratory test to document a previous EBV infection. Antibodies against EBNA-1 develop slowly and usually are not detectable until 90 days or longer after onset of illness. Therefore, the presence of EBNA-1 antibodies during an acute illness rules out acute primary EBV infection. In general, the vast majority of EBV infections can be staged by measuring VCA IgM, VCA IgG and EBNA-1 IgG serum antibodies as shown in Table 1. Early antigen (EA) IgG antibodies are not diagnostic of primary EBV infection because only 60–80% of patients are positive during the acute illness and these antibodies can be found in 20% of healthy individuals.^65^

IgG avidity assays may be useful in cases where staging is unclear after the above tests have been performed.^66, 67^ The principle is that IgG antibodies during the acute phase of infection do not bind to their target as tightly as antibodies produced during convalescence. Low avidity antibodies can be dissociated from their target by exposure to urea or another chaotropic reagent. Antibodies remaining after chaotropic reagent treatment are high avidity antibodies representative of late-stage infection.

## Influence of genetics on susceptibility to and severity of infectious mononucleosis

Hwang *et al.*,^68^ utilizing the California Twin Program registry, reported that concordance for infectious mononucleosis in monozygotic twins was twice that of dizygotic twins. They interpreted their results as ‘compatible with a heritable contribution to the risk of infectious mononucleosis.' Rostgaard *et al.*^69^ extended these findings by tracking familial clustering of hospitalized cases of infectious mononucleosis. Using very large Danish national databases, these investigators reported that same-sex twins had a rate ratio of 9.3 for infectious mononucleosis as compared with 2.3 for first-degree relatives (opposite-sex twins, siblings and parents), 1.4 for second-degree relatives (half-siblings, grandparents, uncles and aunts) and 1.0 for third-degree relatives (first cousins). The 95% confidence intervals for those four classes of relationships did not overlap, supporting the conclusion that degree of relatedness increased the likelihood of contracting the disease.

Twins, unless they are separated at birth, share the same environment and probably have similar behavior, which cannot be ruled out as risk factors. However, the significantly greater number of cases in monozygotic or same-sex twins versus dizygotic or opposite-sex twins in both the California and the Danish studies is compelling evidence that susceptibility to infectious mononucleosis has a genetic component.

## Sequelae: Hodgkin lymphoma and multiple sclerosis

EBV infection (either symptomatic or asymptomatic) has been associated with a farrago of neoplastic and autoimmune conditions as reviewed by Odumade *et al.*^70^ In terms of symptomatic EBV infection, a history of infectious mononucleosis is a strong risk factor for Hodgkin lymphoma,^71^ as well as for multiple sclerosis.^72^ The reason why these diseases and symptomatic primary EBV infection are linked is not known. A plausible explanation well worth exploring is that host genetic and/or environmental factors for severity of primary EBV infection and Hodgkin lymphoma or multiple sclerosis are the same.

## Prevention and treatment of infectious mononucleosis

Development of a prophylactic EBV vaccine has been a priority for researchers in the field ever since the idea was suggested by Epstein and Achong in 1973.^73^ Progress has been painfully slow. The first phase 1 trial for a prophylactic EBV vaccine was not reported until 1995,^74^ and results of the first phase 2 study were not published until 2007.^75^ To date, two prophylactic vaccine constructs have been tested in humans: subunit gp350 and an EBNA-3A peptide.^75, 76^ EBV vaccines have been recently reviewed in this journal.^77^

There is no approved treatment for infectious mononucleosis. Several nucleoside analogs have *in vitro* activity against EBV,^78^ but a clinical benefit has not yet been proven for any of them. Valacyclovir is worth mentioning because it is generic and has very few side effects. We compared valacyclovir (3 g day^−1^ for 2 weeks) with no antiviral therapy in a group of 20 university undergraduate students with acute infectious mononucleosis. The proportion of valacyclovir recipients versus control subjects who had ⩾2 log_10_ decrease in EBV copies was significantly greater for both the oral wash fluid-derived cell pellet (*P*=0.03) and supernatant (*P*=0.001) samples. At the end of the treatment period, the number of reported symptoms (*P*=0.03) and the severity illness (*P*=0.05) were significantly reduced among valacyclovir recipients as compared with controls. As our study contained few subjects and was not placebo controlled, these results must be confirmed in a larger, placebo-controlled trial.^79^

Corticosteroids are often prescribed to treat inflammatory complications such as airway obstruction or autoimmune phenomena such as anemia and thrombocytopenia, but the value of these drugs is controversial and they may impair viral clearance.^80^

## Future research goals

The highest priority in our opinion is development of an EBV vaccine. EBV vaccine has the potential to prevent or modify the severity of infectious mononucleosis, multiple sclerosis, EBV-positive Hodgkin lymphoma, endemic Burkitt lymphoma and nasopharyngeal carcinoma among other entities.^81, 82^

A second goal is to define the genetic, immunologic and/or environmental factors that affect disease severity and propensity to develop EBV-spurred cancer or autoimmune disease. As part of this endeavor, studies should be directed at the question, ‘Why is primary EBV infection more likely to cause infectious mononucleosis in adolescents and young adults?'

A third goal is to discover specific anti-EBV drugs to treat infectious mononucleosis. In this regard, the field of anti-cytomegalovirus drug development is much further along than that of EBV.^83^

## Figures and Tables

**Table 1:** Staging EBV infection by enzyme immunoassay antibody results

*Stage of infection*	*Time after onset of illness*	*VCA IgM*	*VCA IgG*	*EBNA-1 IgG*
EBV naive	—	Negative	Negative	Negative
Acute primary infection	0–3 Weeks	Positive	Negative or positive	Negative
Subacute infection	4 Weeks–3 months	Positive	Positive	Negative
Convalescent infection	4–6 Months	Negative or positive	Positive	Negative or positive
Past infection	>6 Months	Negative	Positive	Positive

Abbreviations: EBNA, EBV nuclear antigen; EBV, Epstein–Barr virus; IgM, immunoglobulin M; VCA, VCA, viral capsid antigen.
